# Three-dimensional Imaging Methods for Quantitative Analysis of Facial Soft Tissues and Skeletal Morphology in Patients with Orofacial Clefts: A Systematic Review

**DOI:** 10.1371/journal.pone.0093442

**Published:** 2014-04-07

**Authors:** Mette A. R. Kuijpers, Yu-Ting Chiu, Rania M. Nada, Carine E. L. Carels, Piotr S. Fudalej

**Affiliations:** 1 Department of Orthodontics and Craniofacial Biology, Radboud University Medical Centre, Nijmegen, the Netherlands; 2 Cleft Palate Craniofacial Unit, Radboud University Medical Centre, Nijmegen, The Netherlands; 3 Department of Dentistry and Craniofacial Center, Chang Gung Memorial Hospital, Taipei, Taiwan; 4 Department of Orthodontics and Dentofacial Orthopedics, University of Bern, Bern Switzerland; 5 Department of Orthodontics, Palacky University Olomouc, Olomouc, Czech Republic; Instituto Butantan, Brazil

## Abstract

**Background:**

Current guidelines for evaluating cleft palate treatments are mostly based on two-dimensional (2D) evaluation, but three-dimensional (3D) imaging methods to assess treatment outcome are steadily rising.

**Objective:**

To identify 3D imaging methods for quantitative assessment of soft tissue and skeletal morphology in patients with cleft lip and palate.

**Data sources:**

Literature was searched using PubMed (1948–2012), EMBASE (1980–2012), Scopus (2004–2012), Web of Science (1945–2012), and the Cochrane Library. The last search was performed September 30, 2012. Reference lists were hand searched for potentially eligible studies. There was no language restriction.

**Study selection:**

We included publications using 3D imaging techniques to assess facial soft tissue or skeletal morphology in patients older than 5 years with a cleft lip with/or without cleft palate. We reviewed studies involving the facial region when at least 10 subjects in the sample size had at least one cleft type. Only primary publications were included.

**Data extraction:**

Independent extraction of data and quality assessments were performed by two observers.

**Results:**

Five hundred full text publications were retrieved, 144 met the inclusion criteria, with 63 high quality studies. There were differences in study designs, topics studied, patient characteristics, and success measurements; therefore, only a systematic review could be conducted. Main 3D-techniques that are used in cleft lip and palate patients are CT, CBCT, MRI, stereophotogrammetry, and laser surface scanning. These techniques are mainly used for soft tissue analysis, evaluation of bone grafting, and changes in the craniofacial skeleton. Digital dental casts are used to evaluate treatment and changes over time.

**Conclusion:**

Available evidence implies that 3D imaging methods can be used for documentation of CLP patients. No data are available yet showing that 3D methods are more informative than conventional 2D methods. Further research is warranted to elucidate it.

**Systematic review registration:**

International Prospective Register of Systematic Reviews, PROSPERO CRD42012002041

## Introduction

Patients with cleft lip and palate (CLP) are treated for an extended period of time. They often undergo several types of surgery as well as other treatment procedures by specialists collaborating with interdisciplinary teams from infancy until adulthood. The surgical procedures are necessary to reconstruct the anatomy of the alveolar arch and the face, and to restore the functions of the palate, lip muscles, and nose. Although treatment improves function and esthetics, it potentially can lead to tissue distortion and have a negative effect on craniofacial growth [1]. This may lead to less optimal facial esthetics along with negative psychosocial effects on a patient’s well-being [2], [3].

Many treatment protocols exist for the management of patients with CLP. Therefore, evaluating the results of treatment becomes more and more important. The Eurocleft study [4] evaluated treatment outcomes in Europe in the 1990s and recently the Americleft study [5], [6]–[9] examined treatment outcome in the US. Both studies proposed documentation and record taking for evaluation of treatment outcomes at certain time points, while they leave liberty for records at other time points. For record taking it appears that the first most complete data records are generally not documented earlier than age 5 [4], [5]. At this age, some records, especially dental casts, have a predictive value for growth and further treatment [10], [11].

It is expected that the majority of cleft palate treatment teams will use newly introduced three dimensional (3D) imaging technology to assess their treatment results. An increasing number of papers have been published regarding 3D evaluation of facial morphology and treatment outcomes in patients with clefts. Pharyngeal space is assessed with magnetic resonance imaging (MRI), computed tomography (CT), or cone beam computed tomography (CBCT). Results of bone grafting are evaluated with CT or CBCT. The jaw relationship, dental and alveolar arch, and the effects of surgery are examined with digital models and CBCT. The guidelines derived from Eurocleft, and later from Americleft, are still based on two-dimensional (2D) evaluation, except for dental casts, which are 3D by nature. Further evaluation may be needed to determine whether guidelines are necessary for the newer craniofacial imaging technologies.

A recent systematic review [12] about methods to quantify soft-tissue based facial growth and treatment outcomes in children younger than 6 years of age concluded that stereophotogrammetry seems to be the best method to longitudinally assess facial growth in these children. Studies on infants with CLP using 3D imaging techniques have been performed mainly to evaluate lip changes after surgery [13]–[15] and the effect of nasoalveolar molding [16].

A systematic review of existing 3D technologies for assessing treatment outcome in patients with CLP would provide clues for evaluating treatment effects and planning, as well as a comparison of treatment possibilities. Therefore, the objective of this systematic review was to identify 3D imaging methods that enable a quantitative analysis of facial soft tissues, velopharyngeal function and airway, skeletal morphology, and dentition in patients with cleft lip and palate.

## Methods

### Protocol and Registration

Inclusion criteria and methods of analysis were specified in advance and registered as a protocol in the International Prospective Register of Systematic Reviews, PROSPERO (http://www.crd.york.ac.uk/Prospero/). The registration number is: CRD42012002041. The protocol for this systematic review and supporting PRISMA checklist are available as supporting information; see Checklist S1 and Protocol S1.

### Eligibility Criteria

Primary publications eligible for inclusion were those using 3D imaging techniques for assessing facial soft tissue or skeletal morphology in CLP patients. Further inclusion criteria were 1) cleft lip with or without cleft palate; 2) sample size larger than 10 for at least one cleft type; 3) patients 5 years of age or older; and 4) publications with quantitative assessment. Patients 5 years and older were included, because it appears that the first most complete data records are generally not documented earlier than age 5 [4], [5]. Exclusion criteria were: 1) craniofacial syndromes; 2) imaging only of neurocranium; 3) injury and trauma; 4) use of only 2D imaging techniques; and 5) reviews, expert opinions, letters, and case reports.

No restrictions were made for language, publication date, and publication status.

### Information Resources

To identify publications, literature was searched until September 2012 using PubMed (1948–2012), EMBASE (1980–2012), Scopus (2004–2012), Web of Science (1945–2012), and the Cochrane Library. The last search was performed September 30th, 2012. Reference lists of identified manuscripts were then hand searched for potentially eligible studies. Digital full text publications were retrieved from licensed digital publishers and paper publications were retrieved from the university library. Authors were contacted when publications could not be retrieved. Gray literature was not searched.

### Search Strategy

A search strategy and list of terms were developed and databases were selected with the help of a senior librarian specialized in health sciences. Medical subject headings and text words in the title and abstract were used for the search strategy in PubMed (Table 1) and search strategies for other databases were derived from this approach.

**Table 1:** PubMed search strategy.

(((((((((4D[tiab] OR 4-dimensional[tiab])) OR (Four Dimensional Computed Tomography[tiab]))) OR (((((Tomography, X-Ray Computed[Mesh] OR Tomography, X-Ray Computed[tiab])) OR (Computed Tomographic[tiab] OR CT[tiab] OR volumetric CT[tiab])) OR (Cone Beam Computed Tomography[tiab] OR CBCT[tiab] OR Spiral Cone Beam Computed Tomography[tiab])) OR (Four Dimensional Computed Tomography[tiab]))) OR (((Photogrammetry[Mesh] OR Photogrammetry[tiab])) OR (stereophotogrammetr*[tiab]))) OR (((((computed tomography[tiab])) OR (computer assisted tomography[tiab]))) OR (((((Tomography, X-Ray Computed[Mesh] OR Tomography, X-Ray Computed[tiab])) OR (Computed Tomographic[tiab] OR CT[tiab] OR volumetric CT[tiab])) OR (Cone Beam Computed Tomography[tiab] OR CBCT[tiab] OR Spiral Cone Beam Computed Tomography[tiab])) OR (Four Dimensional Computed Tomography[tiab])))) OR (((Magnetic Resonance Imaging[Mesh] OR Magnetic Resonance Imaging[tiab] OR Magnetic Resonance Image[tiab] OR Magnetic Resonance Images[tiab])) OR (MRI[tiab]))) OR (((((Imaging, Three-Dimensional[Mesh] OR Imaging, Three-Dimensional[tiab])) OR (3D[tiab] OR three dimensional[tiab])) OR (3D[tiab] AND (image[tiab] OR images[tiab] OR imaging[tiab]))) OR (3D image[tiab] OR 3D images[tiab] OR 3D imaging[tiab]))) AND ((((cleft lip[Mesh] OR cleft lip[tiab])) OR (cleft palate[Mesh] OR cleft palate[tiab])) OR ((((CLP[tiab])) OR (UCLP[tiab])) OR (BCLP[tiab])))

The terms used in the search strategy were:

- 1- *Concerning cleft lip and palate*: Cleft lip, cleft palate, CLP, UCLP, BCLP
- 2-*Three dimensional*: Imaging three-dimensional, 3D, three dimensional, image, images, imaging, 3D image, 3D images, 3D imaging
- 3- *CT*: Tomography, X-ray computed, computed tomographic, CT, volumetric CT, computed tomography, computer assisted tomography
- 4- *CBCT*: Cone beam computed tomography, CBCT, spiral cone beam computed tomography
- 5- *Photos*: Photogrammetry, stereophotogrammetr*
- 6- *MRI:* Magnetic resonance imaging, magnetic resonance image*, MRI
- 7- *4D:* 4D, 4-dimensional, four dimensional computed tomography
- 8- *Ultrasound:* Ultrasonography, echography

The title and abstract of studies were first independently screened by two reviewers (YC and MK). The reviewers were chosen based on their experience of 3D-techniques and cleft lip and palate treatment. Disagreements were resolved by discussion and consensus. After review of only the abstracts, they were scored as” included”, “excluded”, or “unclear”. Then, the full text was retrieved for included articles and articles with unclear abstracts. Full text assessments were performed independently by the same two reviewers. Disagreements were resolved by discussion and consensus. All studies were categorized by the method of imaging used.

### Quality Assessment

The included studies were evaluated according to the quality assessment instrument used by Gordon et al [17]. This instrument includes an assessment of study bias and criteria, as shown in Table 2. Two reviewers utilized the quality assessment instrument (QAI) independent of each other (MK and YC). After that, disagreements were resolved by discussion and consensus. When no consensus could be reached, a senior researcher (PF) experienced with this QAI and also familiar with cleft lift and palate treatment made the final decision.

**Table 2:** Quality assessment instrument.

**I. Study design (7** 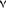 **)**
A. Objective–objective clearly formulated ( 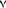 )
B. Sample size–considered adequate ( 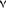 )
C. Sample size–estimated before collection of data ( 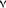 )
D. Selection criteria–clearly described ( 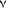 )
E. Baseline characteristics–similar baseline characteristics ( 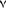 )
F. Timing–prospective ( 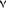 )
G. Randomization–stated ( 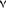 )
**II. Study measurements (3** 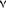 **)**
H. Measurement method–appropriate to the objective ( 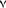 )
I. Blind measurement–blinding ( 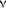 )
J. Reliability–adequate level of agreement ( 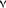 )
**III. Statistical analysis (5** 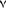 **)**
K. Dropouts–dropouts included in data analysis ( 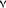 )
L. Statistical analysis–appropriate for data ( 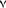 )
M. Confounders–confounders included in analysis ( 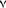 )
N. Statistical significance level–*P* value stated ( 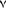 )
O. Confidence intervals provided ( 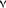 )Maximum number of 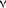 s = 15

(Gordon JM, Rosenblatt M, Witmans M, Carey JP, Heo G, Major PW, et al. Rapid palatal expansion effects on nasal airway dimensions as measured by acoustic rhinometry. A systematic review. *Angle Orthod*. 2009;79(5): 1000–1007.).

A checkmark was scored when a criterion was fulfilled. Depending on the study design, a maximum of 15 criteria could be scored. When certain criteria were not applicable for the study design, less than 15 criteria were scored and the non-applicable criteria were not used for assessing the overall study quality. Study quality was expressed as the number of criteria fulfilled divided by the total number of applicable criteria multiplied by 100. The studies were grouped according to the method of imaging. In cases where criteria were not applicable to the study design, the scoring was marked with a dot. Arbitrarily, a cut-off of 60% or higher was graded (after evaluation of the data) as good quality and below 60% was graded as poor quality [18].

### Statistics

Cohens’s kappa statistics were used to assess the inter-observer reliability of the selection of articles based on the full text. The inter-rater reliability of the quality assessment was calculated using kappa statistics on 23 randomly selected articles scored by two reviewers (MK and YC). The strength of agreement was defined according to Landis and Koch [19]: poor (kappa <0.20), fair (kappa = 0.21–0.40), moderate (kappa = 0.41–0.60), good (kappa = 0.61–0.80), and very good (kappa = 0.81–1.00). Fisher’s exact test was performed to test for differences in quality between groups of methods with a cut-off score of 60% for the QAI. SPSS version 19.0 was used.

## Results

### Study Selection

**Figure 1:**
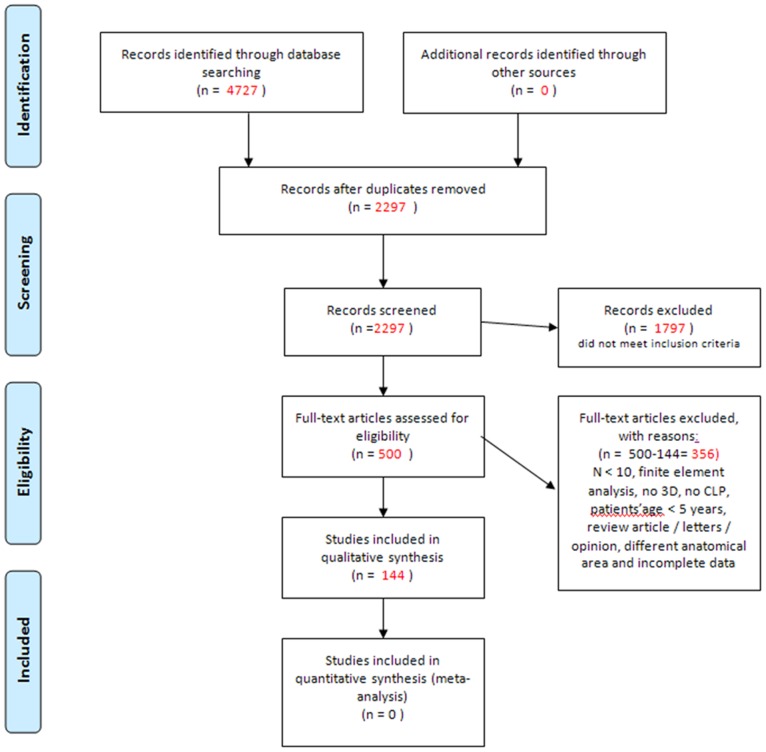
PRISMA flow chart of the study selection process.

The inter-observer kappa for the reliability of study selection based on the full text was 0.76, which qualified as good [19]. The searches in PubMed, EMBASE, Cochrane Library, Web of Science, and Scopus yielded a total of 4727 citations and the hand search provided no additional publications. After adjusting for duplicates, the title and abstract of 2297 citations were screened. After this screening, 1797 articles were excluded because they did not meet the inclusion criteria. The full text was assessed for the 500 remaining articles. All of these articles were retrieved. All, except 2, articles were available in e-journals. Two articles were retrieved by contacting the author. Reasons for excluding studies after full text assessment were: different anatomical region; articles were letters, opinions, or reviews; and the studies applying finite element models. A total of 144 studies met the inclusion criteria. The PRISMA flow diagram is shown in Figure 1. Of the 144 included studies for this review, 49 used CT as a 3D imaging modality, 23 used CBCT, 21 studies involved 3D stereophotogrammetry, 26 studies used laser surface scanning (including n = 5 3D digital dental casts), 7 used MRI, and another method of 3D analysis was used in 18 studies [10], [11], [20]–[159].

### Quality Assessment of Studies

The inter-rater reliability for all 15 criteria of the QAI were between −0.42 and 1 (inter-observer kappa). The kappa’s for the different criteria (A to O) were: A = 1; B = 0.76; C = 1; D = 0; E = 0.39; F = 0.6; G = 0.52; H = −0.42; I = 0.28; J = 0.48; K = 0.64; L = 0.34; M = 0.67; N = 0.73; and O = 0.46. Eight of 15 criteria had a kappa of 0.50 or higher. The inter-rater reliability for criteria D (selection criteria – clearly described) and H (measurement method – appropriate to the objective) were below 0.20 with the reviewers disagreeing on 3 out of 23 articles.

The assessment of the methodological quality of all reviewed studies resulted in scores ranging from 8% to 92%. Of the 144 included studies, 63 (43.8%) qualified as good according to a methodological quality score ≥60% (Tables 3 to 8). Complete score summaries for the different imaging techniques are shown in Tables S1 to S6. Fisher’s exact test (*p* = 0.232) showed no statistically significant difference in the number of studies with good methodological quality among the groups of methods. The numbers of good (score >60%) and low quality studies were comparable for each method.

**Table 3:** Methodological quality scores of CT studies with an overall quality score of ≥60%.

First author	Year	Topic	Study design							Measure			Statistics					Score
			A	B	C	D	E	F	G	H	I	J	K	L	M	N	O	
Ras	1997	sagittal position maxilla	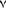	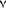	o	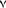	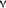	o	.	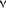	.	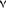	.	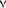	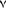	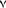	o	75%
Van der Meij	2001	bone graft quantity	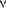	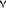	o	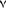	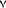	o	.	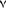	.	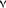	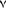	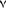	o	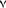	o	69%
Kawakami	2003	bone graft height, density	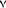	o	o	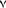	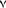	o	.	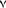	.	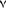	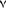	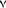	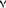	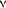	o	69%
Van der Meij	2003	bone graft	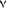	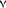	o	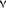	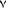	o	.	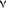	.	o	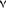	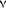	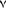	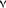	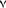	77%
Kita	2004	need bone graft	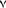	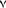	o	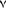	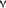	o	.	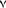	.	o	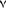	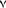	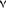	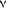	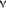	77%
Schliephake	2006	maxillary arch width	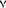	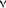	o	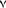	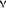	o	.	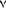	.	o	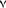	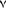	o	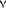	o	62%
Kim	2008	bone graft size, volume	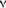	o	o	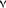	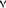	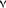	.	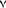	.	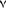	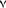	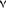	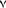	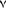	o	77%
Suri	2008	midface, maxilla	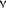	o	o	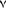	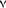	o	.	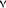	.	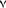	.	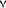	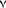	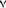	o	66%
Alonso	2010	bone graft size, volume	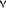	o	o	o	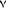	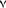	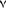	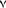	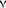	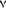	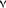	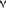	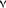	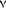	o	73%
Saijo	2010	ossification pal suture	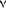	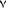	o	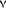	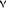	o	.	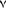	.	o	.	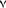	.	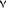	.	70%
Lee	2011	pterygomaxillary region	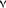	o	o	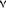	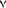	o	.	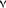	o	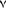	.	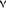	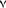	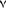	o	62%
Li	2011	maxilla	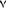	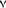	o	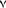	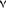	o	.	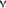	.	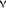	.	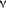	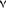	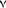	o	75%
Tulunoglu	2011	cephalometry 2d vs 3D	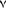	o	o	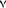	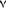	o	.	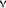	.	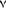	.	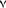	.	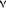	o	63%
Choi	2012	ossification pal suture	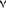	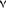	o	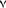	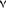	o	.	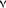	.	o	.	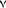	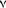	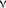	o	67%
Seike	2012	bone graft size, density	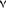	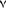	o	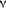	.	o	.	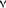	.	o	.	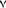	.	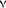	o	60%
Ye	2012	palatal shelf elevation	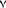	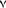	o	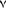	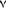	o	.	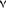	.	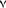	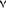	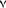	o	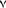	o	69%

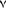
 = Fulfilled satisfactorily the methodological criteria;

o = Did not fulfill the methodological criteria;

. = Not applicable.

**Table 4:** Methodological quality scores of CBCT studies with an overall quality score of ≥60%.

First author	Year	Topic	Study design							Measure			Statistics					Score
			A	B	C	D	E	F	G	H	I	J	K	L	M	N	O	
Dickinson	2008	bone graft	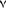	o	o	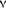	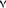	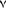	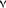	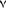	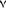	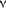	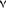	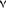	.	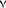	o	79%
Nagasao	2008	nasal septum	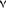	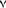	o	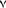	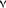	o	.	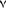	.	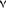	.	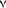	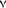	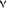	o	75%
Oberoi	2009	bone graft volume	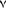	o	o	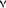	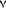	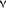	.	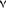	.	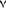	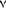	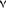	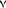	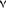	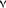	85%
Oberoi	2010	canine	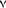	o	o	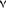	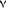	o	.	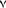	.	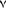	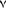	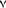	.	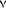	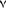	75%
Shirota	2010	bone graft volume	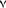	o	o	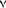	.	o	.	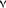	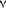	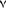	.	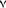	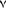	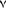	o	67%
Li, F.	2011	maxilla	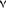	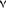	o	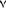	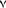	o	o	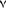	.	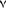	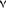	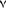	.	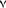	o	69%
Veli	2011	Mandible	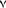	o	o	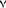	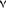	o	.	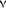	o	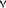	.	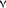	.	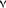	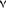	67%
Leenarts	2012	dental arches (Goslon)	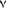	o	o	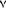	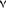	o	.	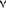	.	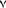	.	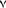	.	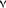	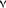	73%
Li	2012	Nose	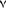	o	o	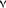	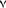	o	.	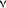	.	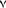	.	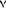	.	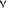	o	63%
Trindade-Suedam	2012	bone graft	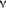	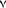	o	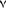	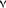	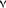	.	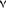	.	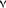	.	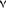	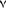	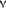	o	83%
Zhou	2013	tooth length	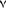	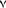	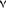	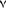	o	o	.	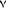	o	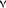	.	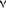	o	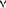	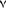	69%

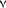
 = Fulfilled satisfactorily the methodological criteria;

o = Did not fulfill the methodological criteria;

. = Not applicable.

**Table 5:** Methodological quality scores of MRI studies with an overall quality score of ≥60%.

First author	Year	Topic	Study design							Measure			Statistics					Score
			A	B	C	D	E	F	G	H	I	J	K	L	M	N	O	
Tian	2010	velopharyngeal space after palatal repair	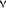	o	o	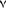	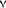	o	.	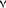	.	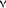	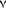	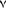	o	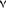	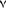	69%
Tian	2010	velopharyngeal motion after palatal repair	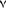	o	o	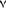	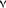	o	.	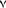	.	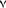	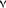	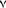	o	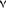	o	62%

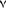
 = Fulfilled satisfactorily the methodological criteria;

o = Did not fulfill the methodological criteria;

. = Not applicable.

**Table 6:** Methodological quality scores of stereophotogrammetry studies with an overall quality score of ≥60%.

First author	Year	Topic	Study design							Measure			Statistics					Score
			A	B	C	D	E	F	G	H	I	J	K	L	M	N	O	
Ras	1994	facial asymmetry	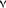	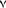	o	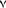	o	o	.	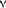	.	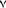	.	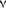	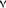	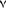	o	67%
Al-Omari	2003	facial deformity scoring	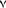	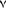	o	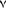	.	o	.	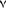	.	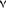	.	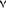	.	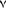	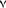	80%
Devlin	2007	nasal symmetry	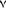	o	o	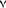	.	o	.	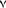	.	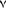	.	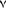	.	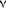	o	60%
Bugaighis	2010	facial shape	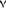	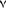	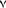	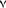	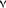	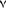	.	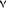	.	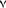	.	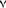	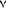	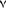	o	92%
Hoefert	2010	soft tissue changes face	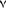	o	o	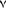	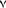	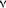	.	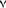	.	o	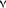	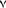	o	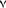	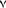	69%
Hoefert	2010	soft tissue changes face	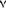	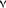	o	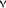	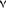	o	.	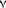	.	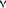	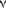	O	.	.	.	70%
Tanikawa	2010	Lips	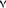	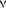	o	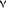	.	o	.	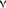	.	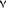	.	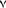	o	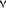	o	64%
Van Loon	2010	Nose	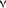	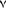	o	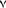	o	o	.	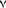	.	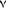	.	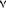	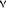	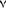	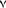	75%
Clark	2011	Lips	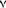	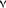	o	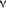	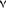	o	.	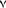	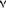	o	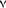	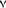	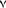	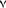	o	71%
Kau	2011	maxilla/lip after bone graft	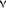	o	o	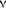	o	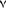	.	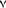	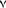	o	.	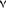	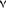	o	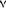	62%
Sander	2011	Nose	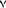	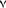	o	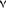	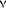	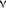	.	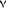	o	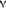	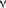	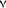	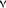	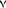	o	79%
Zreaqat	2012	lips, eyes, nose, chin with controls	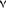	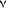	o	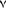	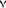	o	.	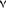	.	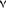	.	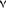	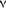	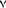	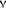	77%
Millar	2013	facial asymmetry and scars	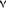	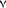	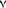	o	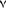	o	.	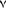	.	o	.	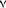	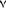	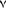	o	67%

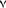
 = Fulfilled satisfactorily the methodological criteria;

o = Did not fulfill the methodological criteria;

. = Not applicable.

**Table 7:** Methodological quality scores of laser surface scanning studies with an overall quality score of ≥60%.

First author	Year	Topic	Study design							Measure			Statistics					Score
			A	B	C	D	E	F	G	H	I	J	K	L	M	N	O	
Bennun	1999	Nose	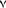	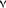	o	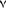	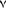	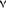	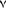	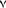	o	O	.	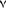	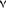	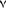	o	71%
Duffy	2000	chin, nose, lips	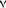	o	o	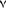	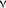	o	.	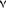	.	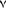	.	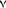	.	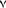	o	64%
Honda	2002	maxillofacial morphology	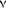	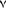	o	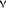	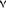	o	.	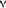	.	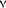	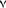	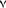	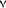	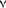	o	77%
Mori	2005	nose, lips	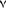	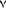	o	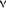	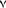	o	.	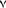	.	O	.	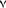	.	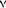	o	64%
Meyer-Marcotty	2009	asymmetry face lay vs specialist	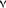	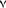	o	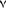	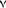	o	.	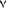	.	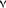	.	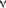	.	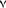	o	73%
Smahel	2009	palatal morph	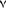	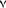	o	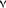	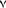	o	.	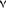	.	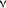	.	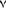	.	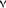	o	73%
Meyer-Marcotty	2010	Face	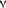	o	o	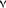	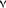	o	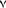	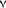	.	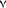	.	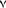	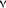	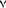	o	69%
Asquith	2012	dental arches (5-yr-olds' index)	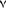	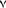	o	o	.	o	.	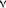	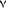	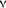	.	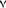	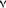	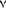	o	67%
Chawla	2012	dental arches (5-yr-olds' index)	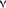	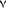	o	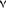	.	o	.	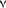	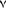	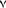	.	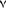	.	.	.	78%
Dogan	2012	dental arches (Goslon)	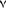	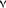	o	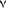	.	o	.	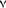	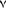	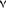	.	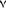	.	.	.	78%
Chawla	2013	dental arches (5-yr-olds' index)	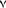	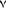	o	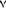	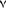	o	.	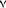	.	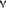	.	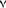	.	.	.	78%

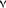
 = Fulfilled satisfactorily the methodological criteria;

o = Did not fulfill the methodological criteria;

. = Not applicable.

**Table 8:** Methodological quality scores of other studies with an overall quality score of ≥60%.

First author	Year	Topic	Study design							Measure			Statistics					Score
			A	B	C	D	E	F	G	H	I	J	K	L	M	N	O	
Kilpelainen	1996	asymmetry palate	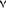	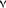	o	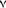	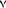	o	.	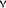	.	o	.	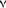	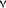	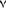	o	67%
Russell	2001	Nose	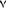	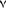	o	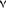	.	o	.	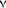	.	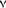	.	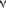	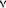	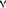	o	73%
Smahel	2003	Palate	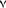	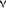	o	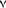	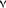	o	.	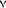	.	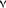	.	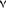	.	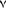	o	73%
Smahel	2004	Palate	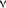	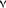	o	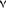	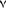	o	.	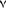	.	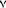	.	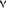	.	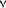	o	73%
Bilwatsch	2006	Nose	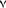	o	o	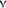	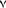	o	.	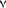	.	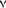	.	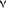	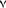	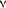	o	67%
Stauber	2008	Nose	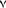	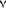	o	o	.	o	.	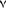	.	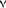	.	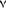	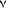	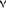	o	64%
Krey	2009	dental arches	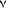	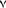	o	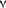	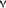	o	.	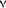	.	o	.	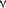	.	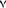	o	64%
Trotman	2010	Lips	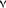	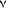	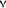	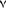	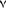	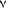	o	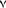	.	.	.	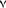	o	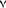	o	75%
Russell	2011	Nose	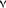	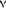	o	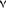	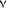	o	.	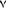	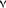	o	.	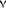	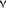	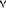	o	69%

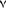
 = Fulfilled satisfactorily the methodological criteria;

o = Did not fulfill the methodological criteria;

. = Not applicable.

CT scanning was the most commonly applied method for 3D imaging of the head in patients with clefts (N = 49 studies; Table 3 and Table S1) [20]–[68]. CT scanning was mainly used to evaluate the results of bone grafting of the alveolar cleft. In addition, the technique was utilized to evaluate bone formation in the palatal cleft, nasal and sinus deformities, and the effects of surgery on the maxilla. The mean methodological score was 54% (range 25–77%). Sixteen (32,7%) of 49 studies [22], [27], [33]–[35], [41], [46], [48], [51], [53], [56]–[58], [61], [64], [66] had a good methodological quality (score of 60% or higher) and the highest score was 77%.

CBCT (N = 23 studies; Table 4 and Table S2) was also used to evaluate the results of bone grafting and to assess the amount of bone needed [69]–[91]. Yet, in the majority of the studies other structures were also assessed such as the pharyngeal airway, canines, alveolar bone adjacent to the cleft, mandible, and nasal morphology. The mean methodological score was 58% (range 18–85%). Of all 23 studies, 11 (47.8%) had a good quality score with the highest score being 85% [73–73,77–79,81,84,85,89,91].

MRI (N = 7 studies; Table 5 and Table S3) was utilized for speech assessments. The velopharyngeal space before and after palatal repair was studied as well as mobility of the lateral pharyngeal wall and the velum [92]–[98]. The mean methodological score was 40% (range 8–69%). The highest quality score was 69% and two studies (28.6%) had good methodological quality [97], [98].

Thirteen (61.9%) [100], [103], [105], [107]–[111], [114], [116], [118], [119] of the 21 studies [99]–[119] using stereophotogrammetry (Table 6 and Table S4) had good quality methodological scores and 92% was the highest score. The mean methodological score was 64% (range 30–92%). Stereophotogrammetry was used for asymmetry assessment of the face, nose, and lips as well as for soft tissue changes after bone grafting or treatment with a Delaire protraction appliance. It was also used for treatment evaluation of lip repair.

Laser scanners (N = 24; Table 7 and Table S5) were used for scanning faces to assess asymmetries and to evaluate changes of the nose, lips, and facial soft tissue before and after surgery [6], [7], [120]–[141]. They were also used to reconstruct digital dental models. The dental models were used to study palatal morphology and dental arch relationships. The dental arch relationship scores on 3D models were compared with plaster cast scores and 2D pictures to evaluate if digital dental models can be used for inter-center studies concerning treatment outcome. The mean methodological score was 58% (range 23–78%). Eleven (45.8%) of 24 studies [6], [7], [122]–[125], [129]–[131], [140], [141] had a good methodological quality and the highest score was 78%.

Various other methods (Table 8 and Table S6) were used that provide 3D coordinates of anatomical structures [142]–[159], like structured lights to create Moiré patterns, reflex microscopy, electromagnetic digitizers, and video tracking. Several studies evaluated palatal morphology, other studies looked at facial asymmetry, nasal asymmetry, and nasal and lip esthetics. One study measured the effect of nasoalveolar molding on the nose [144]. The mean methodological score was 62% (range 36–75%). The highest quality score in this group was 75% and nine of 18 studies (50%) had a good methodological quality [143], [148], [153]–[159].

### Reliability

Scores for reliability and measurement errors of the studies with good methodological quality (score >60%) are shown in Table 9. The majority of the studies reported inter- and intra-rater reliability and the methods used to assess these factors were appropriate for the measurements performed. However, the magnitude of the random error was reported only in a minority of studies.

**Table 9:** Reliability of methods for 3D imaging in cleft lip and palate patients in studies with good methodological quality.

first author	Year	Topic	raters	subjects/objects included in error analysis	duplicate measurements	reliability corr coeff	systematic error determined	Randomerror	weighted kappa
***CT***									
Ras	1997	maxilla, position(mm)	2	17	2		y		
van der Meij	2001	bone graft quantity, surface (mm^2^)	1	1	10			1.95%*	
Kawakami	2003	bone graft density (grading scale)	1	19	2	0.99	y		
van der Meij	2003	bone graft, surface (mm^2^)	–	–	–				
Kita	2004	bone graft, need (grading scale)	2	24	0				
Schliephake	2006	maxillary arch width (mm)	–	–	–				
Kim	2008	bone graft, volume(mm^3^)	1	15	2		y		
			2	15	2		y		
Suri	2008	midface (mm)	1	3	3		y		
Alonso	2010	bone graft, bone fill (%)	1	16	2		y		
Saijo	2010	pal suture, ossification (mm)	–	–	–				
Lee	2011	pterygomaxillary region (mm)	–	10	2		y	0.4	
Li	2011	maxilla (mm)	1	–	2		y		
Tulunoglu	2011	cephalometry 3D (mm, degrees)	1	15	2	0.88–0.99			
Choi	2012	pal suture, ossification (mm)	–	–	–				
Seike	2012	bone graft, size (mm), bone graft, density (mg Calcium)	–	–	–				
Ye	2012	maxillary arch width (mm)	1	30	3	0.84			
***CBCT***									
Dickinson	2008	bone graft (grading system)	3	–	-			1.9%*	
Nagasao	2008	nasal septum (mm)	–	–	–				
Oberoi	2009	bone graft, bone fill (%)	1	5	2	>0.9			
			2	5	2	>0.9			
Oberoi	2010	Canine, eruption (mm)	2	10	2			0.3–1.03	
Shirota	2010	bone graft, volume(cm^3^)	1	13	3				
Li, F	2011	Maxilla, position (mm)	1	20	2	0.98			
Veli	2011	Mandible (mm, mm^3^)	–	15	2		y		
Leenarts	2012	dental arch relationship (1–5 Goslon grading scale)	4	26	2	0.83–0.97	y	0.18–0.45	0.72–0.93
Li	2012	Nose, angles (degrees)	2	16	2	0.98–0.99			
			1	16	2	0.94–0.99			
Trindade–Suedam	2012	bone graft, presence of bone (grading scale)	3	––	2		y		
Zhou	2013	Teeth movement (mm)	1	20	2			2%*	
***MRI***									
Tian	2010	velopharyngeal space(mm)	1	2	2	0.92–0.99			
			2	2	2	0.89–0.98			
Tian	2010	velopharyngeal motion (mm, ratios)	1	6	2	0.92–0.99			
			2	6	2	0.89–0.98			
***Stereophotogrammetry***									
Ras	1994	face, asymmetry (mm)	1	10	4		y		
Al–Omari	2003	face, deformity scoring (rating scale)	10	31	2				0.42–0.72
Devlin	2007	nose, symmetry (mm)	1	1	10			0.46	
Bugaighis	2010	face, shape (mm)	–	–	–			0.5	
Hoefert	2010	face, controls (mm)	1	7	10				
		CLP (mm)	1	22	10				
Hoefert	2010	face (mm)	1	29	10			0.31–0.55	
Tanikawa	2010	lips (mm)	1	10	2				
van Loon	2010	Nose, volume (mm^3^)	1	12	2	0.97–1.00	y	55.68–129.86	
			2	12	2	0.96–1.00	y	56.32–147.40	
Clark	2011	lips (mm)	–	–	–				
Kau	2011	maxilla/lip (mm)	–	–	–				
Sander	2011	Nose (mm)	1	9	3	0.99			
Zreaqat	2012	face (mm)	1	20	2	0.97–0.98			
Millar	2013	facial asymmetry, scars (algorithm score, ratios, scale)	–	–	–				
***Laser surface scanning***									
Bennun	1999	Nose (mm)	–	–	–				
Duffy	2000	chin, nose, lips (mm)	2	16	2			0.47–5.4%*	
Honda	2002	Maxillary dental arch (mm, mm^2^, degrees)	–	–	–				
Mori	2005	nose, lips (mm, degrees)	–	–	–				
Meyer–Marcotty	2009	Face, asymmetry (mm)	–	–	2			<0.006	
Smahel	2009	palate (mm)	–	–	–	>0.98		0.03–2.45	
Meyer–Marcotty	2010	Face (mm)	–	–	2			<0.006	
Chawla	2012	dental arches (1–5grading scale of 5-yr-olds' index)	7	45	2				0.67–0.88
Asquith	2012	dental arches (1–5 grading scale of 5-yr-olds' index)	3	30	2				062–0.83
Dogan	2012	dental arches (1–5 Goslon grading scale)	2	70	3				0.82–0.96
Chawla	2013	dental arches (1–5grading scale of 5-yr-olds' index)	3	45	2				0.74–0.83
***Other***									
Kilpelainen	1996	palate (mm, degrees)	–	–	–				
Russell	2001	Nose (degrees, VAS scale)	6	28	1	0.74			
Smahel	2003	Palate (mm, mm^2^)	1	–	2	>0.95		0.03	
Smahel	2004	Palate (mm, mm^2^)	1	–	2	>0.95		0.03	
Bilwatsch	2006	nose (mm, degrees)	–	22	2		y	<1mm, <1.5^0^	
Stauber	2008	nose (mm, degrees)	–	40	2		y	<1mm, <1.5^0^	
Krey	2009	dental arches (mm)	–	–	–				
Trotman	2010	Lips, distances and movements (mm)	–	–	–				
Russell	2011	Nose (VAS),	6	48	1	0.74			

* = maximum of variable of landmark/distance reproducibility.

## Discussion

The number of publications listed in PubMed on 3D-imaging in CLP patients is steadily rising. A wide variety of different 3D imaging techniques and evaluation methods are used for the craniofacial skeleton and surrounding soft tissues. Below, we discuss the results of this systematic review concerning the 3D-techniques for facial soft tissues, velopharyngeal function and the airway, the craniofacial skeleton, and dentition.

### Soft Tissue Analysis

The majority of the studies concerning soft tissues that had a methodological quality ≥60% were performed with laser surface scanning (Table 6) or stereophotogrammetry (Table 7). However, only a few studies reported the magnitude of the measurement error (Table 9). The maximum reported error for soft tissue measurements with 3D-stereophotogrammetry and laser surface scanning was 0.55 mm [109]. Bilwatsch [155] and Stauber [156] used an optical 3D sensor to acquire facial surface data (Table 8) and they reported a measurement error <1 mm. Only one study reported a measurement error for volume measurements of the nose, with a maximum of 147.40 mm^3^ [111].

Based on the measurement errors in the good quality studies, laser surface scanning and 3D stereophotogrammetry seem to be reliable methods for quantitatively measuring asymmetry and 3-dimensional changes in soft tissues after treatment. For qualitative scoring of asymmetry and esthetics using an expert panel, it is necessary to familiarize the panel members with 3D-stereophotogrammetrical images prior to the scoring task [103]. Dynamic 4D-assessment of soft tissues can register functional repair, but this technique still is in its infancy as only 1 high quality study was found [158].

### Velopharyngeal Function and the Airway

CT and CBCT were used to assess the bony structures of the nose and development of sinuses. Some CBCT and CT studies examined the distances and volumes of the pharyngeal airway space [28], [60], [82], [90]. None of these studies had a high quality score; therefore, we are not able to draw conclusions on the value of CT and CBCT for measuring the airway space in CLP. In two high quality studies, MRI was used to evaluate velopharyngeal function at rest and during phonation, but the random error was not reported [97], [98]. This may indicate that MRI is an adequate, although expensive, technique for measuring the space and motion of the pharyngeal airway.

### Craniofacial Skeleton

CT and CBCT are mainly used for planning orthognathic surgery before and after treatment and for assessing anatomical differences in the nose [47], [56], [79], [81], [85]. These techniques are also used for treatment planning and measuring the results of bone grafting [30], [33]–[35], [46], [64], [73], [75], [77], [78], [89]. Most studies report that no systematic measurement error was present, but the magnitude of the random error was hardly ever reported.

CBCT is a recent radiological technique that became more widely available for imaging the craniofacial region after 2005. CT, which has a much higher radiation dose, was the most commonly used technique for 3D-imaging before CBCT. The SEDENTEXCT Consortium stated, in regards to the radiation dose, that ‘’the application of CBCT in cleft lip and palate patients was found to be the simplest to support’’ in dentistry [160]. They further stated that CBCT may be preferred in situations where CT scanning is currently used for the assessment of cleft lip and palate. The few studies concerning CT or CBCT that reported the reliability showed an acceptable measurement error for both techniques. Therefore, CBCT imaging could be the preferred method for assessing bone volume, as well as for surgical planning, since it has a lower radiation dose than CT scanning. However, further investigation is necessary to determine the influence of this new 3D facial imaging modality on treatment planning, treatment outcome, and treatment evaluation.

### Dentition

Laser surface scanning, CT, CBCT, or moiré photography are used for reconstruction of digital dental casts from plaster casts or from scanning of the impressions [6], [7], [41], [66], [84], [130], [140]–[142], [153], [154], [157]. The majority of these studies reported good reliability. Some studies compared digital models, plaster models, and 2D photographs to assess if digital models can be used to assess outcome and future treatment expectations with the GOSLON yardstick or the 5-year olds’ index [84], [140], [141]. When overlooking the measurement errors in the high quality studies, it seems that digital models obtained with the aid of 3D imaging are a valid alternative for plaster models when assessing treatment outcome with a yardstick as well as for assessment of arch width and palatal morphology.

The dentition has also been studied with CT and CBCT. The bone height of teeth next to the bone graft, eruption, and dental abnormalities have been studied [77], [79], [91] and good reliability was reported. Although the SEDENTEXCT statement [160] includes CLP as one of the few justified reasons for a CBCT in dentistry, there are currently no studies that confirm changes in the diagnosis lead to better treatment planning or outcome in CLP patients when three-dimensional X-rays were used instead of 2D X-rays [18]–[160]. Therefore, the cost benefit of 3D radiology in this situation should be considered.

### Limitations of this Systematic Review

The methodological qualities of the selected articles were assessed according to a scoring system repeatedly used in systematic reviews in orthodontics, which was originally developed by Lagravere [161] and later adapted by Gordon [17]. The method is mainly used for assessing the quality of prospective randomized studies. Only 63 out of 142 studies qualified as being of good methodological quality. The studies were mostly retrospective with relatively small sample sizes and often used descriptive outcome variables. Some criteria used for this study (Table 2), such as the estimation of appropriate sample size before data collection (C), prospective study design (F), randomization (G), and blinding (I), which are all are crucial criteria for high quality studies, were rarely scored as being fulfilled satisfactorily in our systematic review. This was partly due to the patient populations, which make blinding as well as randomization difficult. These were limitations inherent to the scoring system used. Yet, we decided to use this scoring system for the assessment of methodological quality of non-randomized studies [162] as there is no other obvious candidate for assessing these type of studies [162]. Other quality assessment instruments, like the Newcastle-Ottawa scale [162] or Jadad scale [163], [164], used for retrospective studies produce highly arbitrary results [162], [163]. There is still a need for a validated quality assessment instrument that is applicable for a wide range of study designs.

The range of inter-observer kappa values for the quality assessment score was −0.42 to 1.0, indicating strengths of agreement from extremely poor to almost perfect. The low kappa values for criteria D (selection criteria) and H (measurement method) in the quality assessment can be explained by the kappa value being influenced by *trait prevalence.* A single disagreement in scoring between two observers could determine whether the kappa value is 1.0 or 0.0. The absence of adequate instructions for the QAI may lead to different interpretations of the data. In addition, difficulties in interpretation of the data due to its presentation and a lack of information concerning methodology in the published papers may explain the wide range in inter-rater kappa scores.

### Conclusions

CT, CBCT, MRI, stereophotogrammetry, and laser surface scanning are the most frequently used 3D techniques in cleft lip and palate patients. These techniques are mainly used for soft tissue analysis, evaluation of bone grafting, and changes in the craniofacial skeleton. MRI seems to be a reliable, although expensive method to determine velopharyngeal function. Digital dental casts are used to evaluate treatment and changes over time. Available evidence implies that 3D imaging methods can be used for documentations of CLP patients. However, there is no data yet showing that 3D methods are more informative than conventional 2D methods. Further research is warranted to elucidate this and to enable the development of new guidelines for documentation and record taking in cleft lip and palate patients.

## Supporting Information

Table S1 Methodological quality scores of CT studies.
(DOCX)

Table S2 Methodological quality scores of CBCT studies.
(DOCX)

Table S3 Methodological quality scores of MRI studies.
(DOCX)

Table S4 Methodological quality scores of stereophotogrammetry studies.
(DOCX)

Table S5 Methodological quality scores of laser surface scanning studies.
(DOCX)

Table S6 Methodological quality scores of other studies.
(DOCX)

Checklist S1 **PRISMA checklist.**
(DOCX)

Protocol S1 **Protocol for the systematic review as registered in PROSPERO (registration number: CRD42012002041).**
(PDF)
