# Supplementary material for: Three-dimensional Imaging Methods for Quantitative Analysis of Facial Soft Tissues and Skeletal Morphology in Patients with Orofacial Clefts: A Systematic Review
Source: PLoS One. 2014 Apr 7;9(4):e93442. doi: 10.1371/journal.pone.0093442 (PMC3977868; doi:10.1371/journal.pone.0093442)
Supplement: Table S1 — Methodological quality scores of CT studies. (DOCX) [file pone.0093442.s001.docx]

**Table S1.** Methodological quality scores of CT studies

| ***First author*** | ***Year*** | ***Topic*** | ***Study design*** | | | | | | | ***Measure*** | | | ***Statistics*** | | | | | ***Score*** |
| --- | --- | --- | --- | --- | --- | --- | --- | --- | --- | --- | --- | --- | --- | --- | --- | --- | --- | --- |
|  |  |  | **A** | **B** | **C** | **D** | **E** | **F** | **G** | **H** | **I** | **J** | **K** | **L** | **M** | **N** | **O** |  |
| Dado | 1997 | alveolar bone and bonegraft | ۷ | o | o | o | ۷ | o | . | ۷ | . | o | . | ۷ | . | . | . | 44% |
| McCance | 1997 | advancement surgery | ۷ | o | o | ۷ | ۷ | o | . | ۷ | . | o | ۷ | o | o | o | o | 38% |
| Ras | 1997 | sagittal position maxilla | ۷ | ۷ | o | ۷ | ۷ | o | . | ۷ | . | ۷ | . | ۷ | ۷ | ۷ | o | 75% |
| Rosenstein | 1997 | bone graft amount | ۷ | o | o | o | ۷ | o | . | ۷ | . | ۷ | . | ۷ | o | ۷ | o | 50% |
| Santiago | 1998 | bone graft | ۷ | o | o | ۷ | ۷ | o | . | ۷ | o | o | ۷ | ۷ | o | ۷ | o | 50% |
| Denny | 1999 | bone graft | ۷ | ۷ | o | ۷ | ۷ | o | . | ۷ | o | o | ۷ | o | o | o | o | 43% |
| Honma | 1999 | bone graft | ۷ | o | o | o | . | o | . | ۷ | . | ۷ | ۷ | ۷ | o | ۷ | o | 50% |
| Suzuki | 1999 | nasal and sinus deformities | ۷ | ۷ | o | o | o | o | . | ۷ | . | o | . | ۷ | o | ۷ | o | 42% |
| Suzuki | 2000 | sinus development | ۷ | ۷ | o | ۷ | o | o | . | ۷ | . | o | . | ۷ | ۷ | ۷ | o | 58% |
| Tai | 2000 | bone graft | ۷ | o | o | ۷ | ۷ | o | . | ۷ | . | o | ۷ | ۷ | o | ۷ | o | 54% |
| Van der Meij | 2001 | bone graft quantity | ۷ | ۷ | o | ۷ | ۷ | o | . | ۷ | . | ۷ | ۷ | ۷ | o | ۷ | o | 69% |
| Kolbenstvedt | 2002 | bone graft and maxilla | ۷ | o | o | ۷ | . | o | . | ۷ | . | o | . | o | . | . | . | 50% |
| Raphael | 2002 | ossification pal suture | ۷ | ۷ | o | ۷ | ۷ | o | . | ۷ | o | o | . | ۷ | o | ۷ | o | 54% |
| Kawakami | 2003 | bone graft height, density | ۷ | o | o | ۷ | ۷ | o | . | ۷ | . | ۷ | ۷ | ۷ | ۷ | ۷ | o | 69% |
| Van der Meij | 2003 | bone graft | ۷ | ۷ | o | ۷ | ۷ | o | . | ۷ | . | o | ۷ | ۷ | ۷ | ۷ | ۷ | 77% |
| Kita | 2004 | need bone graft | ۷ | ۷ | o | ۷ | ۷ | o | . | ۷ | . | o | ۷ | ۷ | ۷ | ۷ | ۷ | 77% |
| Arctander | 2005 | bone graft | ۷ | o | o | ۷ | ۷ | o | . | ۷ | . | o | . | ۷ | . | ۷ | o | 55% |
| Iino | 2005 | bone graft | ۷ | ۷ | o | ۷ | . | o | . | ۷ | . | o | . | o | o | o | o | 36% |
| Yin | 2005 | ossification pal suture | ۷ | ۷ | o | ۷ | o | o | . | ۷ | . | o | . | ۷ | ۷ | ۷ | o | 58% |
| Chowdhury | 2006 | bone graft | ۷ | ۷ | o | ۷ | o | o | . | o | . | o | . | o | o | o | o | 25% |
| Feichtinger | 2006 | bone graft | ۷ | o | o | o | ۷ | o | . | ۷ | . | o | ۷ | o | . | o | o | 33% |
| Schliephake | 2006 | maxillary arch width | ۷ | ۷ | o | ۷ | ۷ | o | . | ۷ | . | o | ۷ | ۷ | o | ۷ | o | 62% |
| Feichtinger | 2007 | bone graft | ۷ | o | o | ۷ | o | o | . | ۷ | . | o | ۷ | o | o | ۷ | o | 38% |
| Herford | 2007 | bone graft | ۷ | o | o | o | ۷ | o | . | ۷ | ۷ | ۷ | ۷ | ۷ | o | ۷ | o | 57% |
| Ozawa | 2007 | bone graft | ۷ | o | o | o | o | o | . | ۷ | . | o | ۷ | ۷ | ۷ | ۷ | o | 38% |
| Feichtinger | 2008 | bone graft | ۷ | o | o | ۷ | ۷ | o | . | ۷ | . | o | ۷ | ۷ | . | ۷ | o | 58% |
| Kim | 2008 | bone graft size, volume | ۷ | o | o | ۷ | ۷ | ۷ | . | ۷ | . | ۷ | ۷ | ۷ | ۷ | ۷ | o | 77% |
| Nagasao | 2008^a^ | nasal septum | ۷ | ۷ | o | ۷ | ۷ | o | . | ۷ | . | o | . | ۷ | o | ۷ | o | 58% |
| Suri | 2008 | midface, maxilla | ۷ | o | o | ۷ | ۷ | o | . | ۷ | . | ۷ | . | ۷ | ۷ | ۷ | o | 66% |
| Nagasao | 2009^a^ | bone graft | ۷ | o | o | o | ۷ | o | . | ۷ | . | o | . | ۷ | ۷ | ۷ | o | 50% |
| Nagasao | 2009^b^ | bone graft | ۷ | ۷ | o | ۷ | o | o | . | ۷ | . | o | . | ۷ | o | ۷ | o | 50% |
| Alonso | 2010 | bone graft size, volume | ۷ | o | o | o | ۷ | ۷ | ۷ | ۷ | ۷ | ۷ | ۷ | ۷ | ۷ | ۷ | o | 73% |
| Mikoya | 2010 | bone graft | ۷ | ۷ | o | ۷ | ۷ | o | . | o | o | ۷ | . | o | o | o | o | 38% |
| Saijo | 2010 | ossification pal suture | ۷ | ۷ | o | ۷ | ۷ | o | . | ۷ | . | o | . | ۷ | . | ۷ | . | 70% |
| Ye | 2010 | maxillary arch width | ۷ | ۷ | o | ۷ | ۷ | o | . | ۷ | . | o | . | ۷ | o | ۷ | o | 58% |
| Chen | 2011 | maxilla | ۷ | o | o | ۷ | ۷ | o | . | ۷ | . | o | ۷ | ۷ | o | ۷ | o | 54% |
| Lee | 2011 | pterygomaxillary region | ۷ | o | o | ۷ | ۷ | o | . | ۷ | o | ۷ | . | ۷ | ۷ | ۷ | o | 62% |
| Li | 2011 | Maxilla | ۷ | ۷ | o | ۷ | ۷ | o | . | ۷ | . | ۷ | . | ۷ | ۷ | ۷ | o | 75% |
| Tulunoglu | 2011 | cephalometry 2d vs 3D | ۷ | o | o | ۷ | ۷ | o | . | ۷ | . | ۷ | . | ۷ | . | ۷ | o | 63% |
| Agarwal | 2012 | maxilla | ۷ | o | o | ۷ | o | o | . | ۷ | . | o | . | ۷ | o | ۷ | o | 42% |
| Aras | 2012 | nasopharyngeal airway | ۷ | o | o | ۷ | o | o | . | ۷ | . | o | . | ۷ | . | ۷ | o | 45% |
| Choi | 2012 | ossification pal suture | ۷ | ۷ | o | ۷ | ۷ | o | . | ۷ | . | o | . | ۷ | ۷ | ۷ | o | 67% |
| Hegab | 2012 | bone graft | ۷ | o | o | ۷ | o | ۷ | o | ۷ | . | o | o | o | o | ۷ | o | 36% |
| Rychlik | 2012 | bone graft | ۷ | ۷ | o | o | ۷ | ۷ | . | ۷ | . | o | . | o | o | ۷ | o | 50% |
| Seike | 2012 | bone graft size, density | ۷ | ۷ | o | ۷ | . | o | . | ۷ | . | o | . | ۷ | . | ۷ | o | 60% |
| Wu | 2012 | whole face | ۷ | ۷ | o | ۷ | ۷ | o | . | ۷ | . | ۷ | . | o | . | o | o | 55% |
| Ye | 2012 | palatal shelf elevation | ۷ | ۷ | o | ۷ | ۷ | o | . | ۷ | . | ۷ | ۷ | ۷ | o | ۷ | o | 69% |
| Yoshida | 2012 | bone graft | ۷ | o | o | ۷ | ۷ | o | . | ۷ | . | o | . | ۷ | . | ۷ | o | 55% |
| Zhang, Y | 2012 | bone graft | ۷ | ۷ | o | o | o | o | o | o | o | o | o | ۷ | o | ۷ | o | 27% |

۷ = Fulfilled satisfactorily the methodological criteria;

o = Did not fulfil the methodological criteria;

. = Not applicable.
