# Supplementary material for: Three-dimensional Imaging Methods for Quantitative Analysis of Facial Soft Tissues and Skeletal Morphology in Patients with Orofacial Clefts: A Systematic Review
Source: PLoS One. 2014 Apr 7;9(4):e93442. doi: 10.1371/journal.pone.0093442 (PMC3977868; doi:10.1371/journal.pone.0093442)
Supplement: Table S2 — Methodological quality scores of CBCT studies. (DOCX) [file pone.0093442.s002.docx]

**Table S2.** Methodological quality scores of CBCT studies

| ***First author*** | ***Year*** | ***Topic*** | ***Study design*** | | | | | | | ***Measure*** | | | ***Statistics*** | | | | | ***Score*** |
| --- | --- | --- | --- | --- | --- | --- | --- | --- | --- | --- | --- | --- | --- | --- | --- | --- | --- | --- |
|  |  |  | **A** | **B** | **C** | **D** | **E** | **F** | **G** | **H** | **I** | **J** | **K** | **L** | **M** | **N** | **O** |  |
| Yin | 2001 | ossification pal suture | ۷ | o | o | ۷ | ۷ | o | . | ۷ | o | o | . | ۷ | o | o | o | 38% |
| Hamada | 2005 | bone graft | ۷ | o | o | o | . | o | . | ۷ | . | o | . | o | o | o | o | 18% |
| Korbmacher | 2007 | cranium | ۷ | o | o | ۷ | . | o | . | ۷ | . | o | . | ۷ | . | ۷ | o | 50% |
| Miyamoto | 2007 | nose | ۷ | ۷ | o | ۷ | o | o | . | ۷ | . | o | . | ۷ | o | ۷ | o | 50% |
| Dickinson | 2008 | bone graft | ۷ | o | o | ۷ | ۷ | ۷ | ۷ | ۷ | ۷ | ۷ | ۷ | ۷ | . | ۷ | o | 79% |
| Nagasao | 2008^b^ | nasal septum | ۷ | ۷ | o | ۷ | ۷ | o | . | ۷ | . | ۷ | . | ۷ | ۷ | ۷ | o | 75% |
| Oberoi | 2009 | bone graft volume | ۷ | o | o | ۷ | ۷ | ۷ | . | ۷ | . | ۷ | ۷ | ۷ | ۷ | ۷ | ۷ | 85% |
| Miyamoto | 2010 | nose | ۷ | o | o | ۷ | ۷ | o | . | ۷ | . | o | . | ۷ | . | ۷ | o | 55% |
| Oberoi | 2010 | canine | ۷ | o | o | ۷ | ۷ | o | . | ۷ | . | ۷ | ۷ | ۷ | . | ۷ | ۷ | 75% |
| Shirota | 2010 | bone graft volume | ۷ | o | o | ۷ | . | o | . | ۷ | ۷ | ۷ | . | ۷ | ۷ | ۷ | o | 67% |
| Li, F. | 2011 | teeth | ۷ | ۷ | o | ۷ | ۷ | o | o | ۷ | . | ۷ | ۷ | ۷ | . | ۷ | o | 69% |
| Contreras | 2011 | alveolar bone cleft | ۷ | o | o | ۷ | . | o | . | ۷ | . | o | . | o | . | ۷ | o | 33% |
| Veli | 2011 | mandible | ۷ | o | o | ۷ | ۷ | o | . | ۷ | o | ۷ | . | ۷ | . | ۷ | ۷ | 67% |
| Chueng | 2012 | pharyngeal airway | ۷ | o | o | ۷ | ۷ | o | . | ۷ | . | ۷ | . | ۷ | o | ۷ | o | 58% |
| Garib | 2012 | alveolar bone thickness | ۷ | o | o | ۷ | ۷ | . | . | ۷ | . | ۷ | . | o | o | o | o | 45% |
| Leenarts | 2012 | dental arches (Goslon) | ۷ | o | o | ۷ | ۷ | o | . | ۷ | . | ۷ | . | ۷ | . | ۷ | ۷ | 73% |
| Li | 2012 | nose | ۷ | o | o | ۷ | ۷ | o | . | ۷ | . | ۷ | . | ۷ | . | ۷ | o | 63% |
| Miyamoto | 2012 | nose | ۷ | o | o | o | ۷ | o | . | ۷ | . | o | ۷ | ۷ | . | ۷ | o | 50% |
| Padricelli | 2012 | teeth | ۷ | o | o | ۷ | o | o | . | ۷ | . | o | . | o | . | o | o | 27% |
| Quereshy | 2012 | bone graft | ۷ | o | o | ۷ | . | o | . | o | ۷ | ۷ | . | o | . | o | o | 36% |
| Trindade-Suedam | 2012 | bone graft | ۷ | ۷ | o | ۷ | ۷ | ۷ | . | ۷ | . | ۷ | . | ۷ | ۷ | ۷ | o | 83% |
| Yoshihara | 2012 | pharyngeal airway morphology | ۷ | o | o | ۷ | ۷ | o | . | ۷ | o | ۷ | . | ۷ | o | ۷ | o | 54% |
| Zhou | 2013 | tooth length | ۷ | ۷ | ۷ | ۷ | o | o | . | ۷ | o | ۷ | . | ۷ | o | ۷ | ۷ | 69% |

۷ = Fulfilled satisfactorily the methodological criteria;

o = Did not fulfill the methodological criteria;

. = Not applicable.
