# Supplementary material for: Three-dimensional Imaging Methods for Quantitative Analysis of Facial Soft Tissues and Skeletal Morphology in Patients with Orofacial Clefts: A Systematic Review
Source: PLoS One. 2014 Apr 7;9(4):e93442. doi: 10.1371/journal.pone.0093442 (PMC3977868; doi:10.1371/journal.pone.0093442)
Supplement: Table S3 — Methodological quality scores of MRI studies. (DOCX) [file pone.0093442.s003.docx]

**Table S3.** Methodological quality scores of MRI studies

| **First author** | **Year** | **Topic** | **Study design** | | | | | | | **Measure** | | | **Statistics** | | | | | **Score** |
| --- | --- | --- | --- | --- | --- | --- | --- | --- | --- | --- | --- | --- | --- | --- | --- | --- | --- | --- |
|  |  |  | **A** | **B** | **C** | **D** | **E** | **F** | **G** | **H** | **I** | **J** | **K** | **L** | **M** | **N** | **O** |  |
| Yamawaki | 1999 | lat pharyngeal wall | ۷ | o | o | o | o | o | . | ۷ | . | o | . | ۷ | o | ۷ | o | 33% |
| Ozgur | 2000 | velum | ۷ | o | o | ۷ | ۷ | o | . | ۷ | . | o | . | o | o | o | o | 33% |
| Vadodaria | 2000 | lat pharyngeal wall | ۷ | ۷ | o | o | o | o | . | ۷ | o | o | . | o | . | . | o | 27% |
| Sehhati | 2006 | med pterygoid muscle | ۷ | o | o | o | o | o | . | o | . | o | . | o | o | o | o | 8% |
| Atik | 2008 | velopharyngeal space | ۷ | o | o | ۷ | o | o | . | ۷ | . | o | ۷ | ۷ | o | ۷ | o | 46% |
| Tian | 2010^a^ | velopharyngeal space after palatal repair | ۷ | o | o | ۷ | ۷ | o | . | ۷ | . | ۷ | ۷ | ۷ | o | ۷ | ۷ | 69% |
| Tian | 2010^b^ | velopharyngeal motion after palatal repair | ۷ | o | o | ۷ | ۷ | o | . | ۷ | . | ۷ | ۷ | ۷ | o | ۷ | o | 62% |

۷ = Fulfilled satisfactorily the methodological criteria;

o = Did not fulfill the methodological criteria;

. = Not applicable.
