# Supplementary material for: Three-dimensional Imaging Methods for Quantitative Analysis of Facial Soft Tissues and Skeletal Morphology in Patients with Orofacial Clefts: A Systematic Review
Source: PLoS One. 2014 Apr 7;9(4):e93442. doi: 10.1371/journal.pone.0093442 (PMC3977868; doi:10.1371/journal.pone.0093442)
Supplement: Table S4 — Methodological quality scores of stereophotogrammetry studies. (DOCX) [file pone.0093442.s004.docx]

**Table S4.** Methodological quality scores of stereophotogrammetry studies

| **First author** | **Year** | **Topic** | **Study design** | | | | | | | **Measure** | | | **Statistics** | | | | | **Score** |
| --- | --- | --- | --- | --- | --- | --- | --- | --- | --- | --- | --- | --- | --- | --- | --- | --- | --- | --- |
|  |  |  | **A** | **B** | **C** | **D** | **E** | **F** | **G** | **H** | **I** | **J** | **K** | **L** | **M** | **N** | **O** |  |
| Ras | 1994^a^ | facial asymmetry | ۷ | ۷ | o | ۷ | o | o | . | ۷ | . | o | . | ۷ | ۷ | ۷ | o | 58% |
| Ras | 1994^b^ | facial asymmetry | ۷ | ۷ | o | ۷ | o | o | . | ۷ | . | ۷ | . | ۷ | ۷ | ۷ | o | 67% |
| Ras | 1995^c^ | facial asymmetry | ۷ | o | o | ۷ | o | o | . | ۷ | . | ۷ | . | ۷ | o | ۷ | o | 50% |
| Ras | 1995^d^ | changes in facial asymmetry | ۷ | ۷ | o | ۷ | o | o | . | ۷ | . | o | o | ۷ | ۷ | ۷ | o | 54% |
| Al-Omari | 2003 | facial deformity scoring | ۷ | ۷ | o | ۷ | . | o | . | ۷ | . | ۷ | . | ۷ | . | ۷ | ۷ | 80% |
| Proff | 2006 | lips | ۷ | o | o | o | . | o | . | ۷ | . | o | . | ۷ | . | o | o | 30% |
| Devlin | 2007 | nasal symmetry | ۷ | o | o | ۷ | . | o | . | ۷ | . | ۷ | . | ۷ | . | ۷ | o | 60% |
| Grewal | 2009 | facial asymmetries | ۷ | o | o | ۷ | o | o | . | ۷ | . | o | ۷ | ۷ | . | ۷ | o | 50% |
| Bugaighis | 2010 | facial shape | ۷ | ۷ | ۷ | ۷ | ۷ | ۷ | . | ۷ | . | ۷ | . | ۷ | ۷ | ۷ | o | 92% |
| Hoefert | 2010^a^ | soft tissue changes face | ۷ | o | o | ۷ | ۷ | ۷ | . | ۷ | . | o | ۷ | ۷ | o | ۷ | ۷ | 69% |
| Hoefert | 2010^b^ | soft tissue changes face | ۷ | ۷ | o | ۷ | ۷ | o | . | ۷ | . | ۷ | ۷ | o | . | . | . | 70% |
| Tanikawa | 2010 | lips | ۷ | ۷ | o | ۷ | . | o | . | ۷ | . | ۷ | . | ۷ | o | ۷ | o | 64% |
| Van Loon | 2010 | nose | ۷ | ۷ | o | ۷ | o | o | . | ۷ | . | ۷ | . | ۷ | ۷ | ۷ | ۷ | 75% |
| Ayoub | 2011 | lips | ۷ | ۷ | o | ۷ | ۷ | o | . | ۷ | . | ۷ | . | ۷ | o | o | o | 58% |
| Clark | 2011 | lips | ۷ | ۷ | o | ۷ | ۷ | o | . | ۷ | ۷ | o | ۷ | ۷ | ۷ | ۷ | o | 71% |
| Kau | 2011 | maxilla/lip after bone graft | ۷ | o | o | ۷ | o | ۷ | . | ۷ | ۷ | o | . | ۷ | ۷ | o | ۷ | 62% |
| Krimmel | 2011 | upper lip after bone graft | ۷ | o | o | o | . | ۷ | . | ۷ | . | o | ۷ | ۷ | o | ۷ | o | 50% |
| Oh | 2011 | upper lip after bone graft | ۷ | ۷ | o | o | . | o | . | ۷ | . | ۷ | . | ۷ | o | ۷ | o | 55% |
| Sander | 2011 | nose | ۷ | ۷ | o | ۷ | ۷ | ۷ | . | ۷ | o | ۷ | ۷ | ۷ | ۷ | ۷ | o | 79% |
| Zreaqat | 2012 | lips, eyes, nose, chin with controls | ۷ | ۷ | o | ۷ | ۷ | o | . | ۷ | . | ۷ | . | ۷ | ۷ | ۷ | ۷ | 77% |
| Millar | 2013 | facial asymmetry and scars | ۷ | ۷ | ۷ | o | ۷ | o | . | ۷ | . | o | . | ۷ | ۷ | ۷ | o | 67% |

۷ = Fulfilled satisfactorily the methodological criteria;

o = Did not fulfill the methodological criteria;

. = Not applicable.
