# Supplementary material for: Three-dimensional Imaging Methods for Quantitative Analysis of Facial Soft Tissues and Skeletal Morphology in Patients with Orofacial Clefts: A Systematic Review
Source: PLoS One. 2014 Apr 7;9(4):e93442. doi: 10.1371/journal.pone.0093442 (PMC3977868; doi:10.1371/journal.pone.0093442)
Supplement: Table S5 — Methodological quality scores of laser surface scanning studies. (DOCX) [file pone.0093442.s005.docx]

**Table S5.** Methodological quality scores of laser surface scanning studies

| ***First author*** | ***Year*** | ***Topic*** | ***Study design*** | | | | | | | ***Measure*** | | | ***Statistics*** | | | | | ***Score*** |
| --- | --- | --- | --- | --- | --- | --- | --- | --- | --- | --- | --- | --- | --- | --- | --- | --- | --- | --- |
|  |  |  | **A** | **B** | **C** | **D** | **E** | **F** | **G** | **H** | **I** | **J** | **K** | **L** | **M** | **N** | **O** |  |
| Stewart | 1996 | nose | ۷ | o | o | o | o | o | . | ۷ | . | ۷ | ۷ | ۷ | o | ۷ | o | 46% |
| McCance | 1997 | face | ۷ | o | o | o | ۷ | o | . | ۷ | . | o | ۷ | o | o | o | o | 23% |
| Bennun | 1999 | nose | ۷ | ۷ | o | ۷ | ۷ | ۷ | ۷ | ۷ | o | o | . | ۷ | ۷ | ۷ | o | 71% |
| Duffy | 2000 | chin, nose, lips | ۷ | o | o | ۷ | ۷ | o | . | ۷ | . | ۷ | . | ۷ | . | ۷ | o | 64% |
| Honda | 2002 | maxillofac morphology | ۷ | ۷ | o | ۷ | ۷ | o | . | ۷ | . | ۷ | ۷ | ۷ | ۷ | ۷ | o | 77% |
| Mori | 2005 | nose, lips | ۷ | ۷ | o | ۷ | ۷ | o | . | ۷ | . | o | . | ۷ | . | ۷ | o | 64% |
| Nakamura | 2005 | chin | ۷ | o | o | ۷ | ۷ | o | . | ۷ | . | o | ۷ | ۷ | o | ۷ | o | 54% |
| Schwenzer-Zimmerer | 2008^a^ | nose, lips | ۷ | o | o | o | o | o | . | ۷ | . | ۷ | ۷ | o | o | o | o | 31% |
| Schwenzer-Zimmerer | 2008^b^ | nose | ۷ | ۷ | o | o | o | o | . | ۷ | . | o | ۷ | o | o | o | o | 31% |
| Meyer-Marcotty | 2009 | asymmetry face lay vs specialist | ۷ | ۷ | o | ۷ | ۷ | o | . | ۷ | . | ۷ | . | ۷ | . | ۷ | o | 73% |
| Smahel | 2009 | palatal morph | ۷ | ۷ | o | ۷ | ۷ | o | . | ۷ | . | ۷ | . | ۷ | . | ۷ | o | 73% |
| Meyer-Marcotty | 2010 | face | ۷ | o | o | ۷ | ۷ | o | ۷ | ۷ | . | ۷ | . | ۷ | ۷ | ۷ | o | 69% |
| Nakamura | 2010 | nose pre/post op | ۷ | o | o | ۷ | o | o | . | ۷ | . | o | ۷ | o | . | . | . | 40% |
| Vasiliauskas | 2010 | palate | ۷ | ۷ | o | ۷ | . | o | . | ۷ | . | o | . | ۷ | o | ۷ | o | 55% |
| Wojtaszek-Slominska | 2010 | palate | ۷ | ۷ | o | ۷ | ۷ | o | . | ۷ | . | o | . | ۷ | o | ۷ | o | 58% |
| Meyer-Marcotty | 2011 | asymmetry face lay vs specialist | ۷ | ۷ | o | ۷ | ۷ | o | . | ۷ | . | o | . | ۷ | o | ۷ | o | 58% |
| Nakamura | 2011^a^ | nose | ۷ | o | o | ۷ | o | o | . | ۷ | . | o | ۷ | ۷ | o | ۷ | o | 46% |
| Nakamura | 2011^b^ | nose | ۷ | o | o | ۷ | o | o | . | ۷ | . | o | ۷ | ۷ | o | . | . | 45% |
| Okawachi | 2011 | nose | ۷ | o | o | ۷ | o | o | . | ۷ | . | o | ۷ | ۷ | o | ۷ | o | 46% |
| Asquith | 2012 | dental arches (5-yr-olds' index) | ۷ | ۷ | o | o | . | o | . | ۷ | ۷ | ۷ | . | ۷ | ۷ | ۷ | o | 67% |
| Bejdova | 2012 | palate | ۷ | ۷ | o | ۷ | ۷ | o | . | ۷ | . | o | . | ۷ | ۷ | o | o | 58% |
| Chawla | 2012 | dental arches (5-yr-olds' index) | ۷ | ۷ | o | ۷ | . | o | . | ۷ | ۷ | ۷ | . | ۷ | . | . | . | 78% |
| Dogan | 2012 | dental arches (goslon) | ۷ | ۷ | o | ۷ | . | o | . | ۷ | ۷ | ۷ | . | ۷ | . | . | . | 78% |
| Chawla | 2013 | dental arches (5-yr-olds' index) | ۷ | ۷ | o | ۷ | ۷ | o | . | ۷ | . | ۷ | . | ۷ | . | . | . | 78% |

۷ = Fulfilled satisfactorily the methodological criteria;

o = Did not fulfill the methodological criteria;

. = Not applicable.
