# Supplementary material for: Three-dimensional Imaging Methods for Quantitative Analysis of Facial Soft Tissues and Skeletal Morphology in Patients with Orofacial Clefts: A Systematic Review
Source: PLoS One. 2014 Apr 7;9(4):e93442. doi: 10.1371/journal.pone.0093442 (PMC3977868; doi:10.1371/journal.pone.0093442)
Supplement: Table S6 — Methodological quality scores of other studies. (DOCX) [file pone.0093442.s006.docx]

**Table S6.** Methodological quality scores of other studies

| ***First author*** | ***Year*** | ***Topic*** | ***Study design*** | | | | | | | ***Measure*** | | | ***Statistics*** | | | | | ***Score*** |
| --- | --- | --- | --- | --- | --- | --- | --- | --- | --- | --- | --- | --- | --- | --- | --- | --- | --- | --- |
|  |  |  | **A** | **B** | **C** | **D** | **E** | **F** | **G** | **H** | **I** | **J** | **K** | **L** | **M** | **N** | **O** |  |
| Kilpelainen | 1996^a^ | palatal asymmetry | ۷ | ۷ | o | o | ۷ | o | . | ۷ | . | o | . | ۷ | ۷ | ۷ | o | 58% |
| Kilpelainen | 1996^b^ | asymmetry palate | ۷ | ۷ | o | ۷ | ۷ | o | . | ۷ | . | o | . | ۷ | ۷ | ۷ | o | 67% |
| Maull | 1999 | nose | ۷ | o | o | o | o | o | . | ۷ | o | o | ۷ | ۷ | o | ۷ | o | 36% |
| Kratzsch | 2000^a^ | palate (rugae) | ۷ | ۷ | o | ۷ | ۷ | o | . | ۷ | o | o | ۷ | ۷ | o | ۷ | o | 57% |
| Kratzsch | 2000^b^ | palate (rugae) | ۷ | ۷ | o | ۷ | ۷ | o | . | ۷ | . | o | ۷ | ۷ | o | ۷ | o | 57% |
| Trotman | 2000 | nasolabial area | ۷ | o | o | o | o | o | . | ۷ | . | ۷ | . | ۷ | ۷ | ۷ | o | 50% |
| Russell | 2001 | nose | ۷ | ۷ | o | ۷ | . | o | . | ۷ | . | ۷ | . | ۷ | ۷ | ۷ | o | 73% |
| Ferrario | 2003^a^ | face | ۷ | o | o | ۷ | ۷ | o | . | ۷ | . | o | . | o | ۷ | ۷ | o | 50% |
| Ferrario | 2003^b^ | face | ۷ | o | o | ۷ | ۷ | o | . | ۷ | . | o | . | ۷ | ۷ | ۷ | o | 58% |
| Ferrario | 2003^c^ | nose | ۷ | o | o | ۷ | ۷ | o | . | ۷ | . | o | . | ۷ | ۷ | ۷ | o | 58% |
| Ferrario | 2003^d^ | lip | ۷ | o | o | ۷ | ۷ | o | . | ۷ | . | o | . | ۷ | ۷ | ۷ | o | 58% |
| Smahel | 2003 | palate | ۷ | ۷ | o | ۷ | ۷ | o | . | ۷ | . | ۷ | . | ۷ | . | ۷ | o | 73% |
| Smahel | 2004 | palate | ۷ | ۷ | o | ۷ | ۷ | o | . | ۷ | . | ۷ | . | ۷ | . | ۷ | o | 73% |
| Bilwatsch | 2006 | nose | ۷ | o | o | ۷ | ۷ | o | . | ۷ | . | ۷ | . | ۷ | ۷ | ۷ | o | 67% |
| Stauber | 2008 | nose | ۷ | ۷ | o | o | . | o | . | ۷ | . | ۷ | . | ۷ | ۷ | ۷ | o | 64% |
| Krey | 2009 | dental arches | ۷ | ۷ | o | ۷ | ۷ | o | . | ۷ | . | o | . | ۷ | . | ۷ | o | 64% |
| Trotman | 2010 | lips | ۷ | ۷ | ۷ | ۷ | ۷ | ۷ | o | ۷ | . | . | . | ۷ | o | ۷ | o | 75% |
| Russell | 2011 | nose | ۷ | ۷ | o | ۷ | ۷ | o | . | ۷ | ۷ | o | . | ۷ | ۷ | ۷ | o | 69% |

۷ = Fulfilled satisfactorily the methodological criteria;

o = Did not fulfill the methodological criteria;

. = Not applicable.
