# Supplementary material for: Three-dimensional Imaging Methods for Quantitative Analysis of Facial Soft Tissues and Skeletal Morphology in Patients with Orofacial Clefts: A Systematic Review
Source: PLoS One. 2014 Apr 7;9(4):e93442. doi: 10.1371/journal.pone.0093442 (PMC3977868; doi:10.1371/journal.pone.0093442)
Supplement: Protocol S1 — Protocol for the systematic review as registered in PROSPERO (registration number: CRD42012002041). (PDF) [file pone.0093442.s008.pdf]

THE UNIVERSITY of York
Centre for Reviews and Dissemination

NHS National Institute for Health Research

PROSPERO International prospective register of systematic reviews

Review title and timescale

1 Review title
Give the working title of the review. This must be in English. Ideally it should state succinctly the interventions or exposures being reviewed and the associated health or social problem being addressed in the review.
3D imaging methods for quantitative assessment of soft tissue and skeletal morphology in patients with cleft lip and palate

2 Original language title
For reviews in languages other than English, this field should be used to enter the title in the language of the review. This will be displayed together with the English language title.

3 Anticipated or actual start date
Give the date when the systematic review commenced, or is expected to commence.
01/12/2011

4 Anticipated completion date
Give the date by which the review is expected to be completed.
22/09/2012

5 Stage of review at time of this submission
Indicate the stage of progress of the review by ticking the relevant boxes. Reviews that have progressed beyond the point of completing data extraction at the time of initial registration are not eligible for inclusion in PROSPERO. This field should be updated when any amendments are made to a published record.

The review has not yet started ×

Review stage	Started	Completed
Preliminary searches	No	Yes
Piloting of the study selection process	No	Yes
Formal screening of search results against eligibility criteria	No	Yes
Data extraction	No	No
Risk of bias (quality) assessment	No	Yes
Data analysis	Yes	No

Provide any other relevant information about the stage of the review here.
n/a

Review team details

6 Named contact
The named contact acts as the guarantor for the accuracy of the information presented in the register record.
Mette Kuijpers

7 Named contact email
Enter the electronic mail address of the named contact.
m.kuijpers@dent.umcn.nl

8 Named contact address
Enter the full postal address for the named contact.
Department of Orthodontics and Craniofacial Biology Radboud University Nijmegen Medical Centre 309 Tandheelkunde PO Box 9101 6500 HB Nijmegen The Netherlands

9 Named contact phone number
Enter the telephone number for the named contact, including international dialing code.
+31 24 3614005

10 Organisational affiliation of the review

Full title of the organisational affiliations for this review, and website address if available. This field may be completed as 'None' if the review is not affiliated to any organisation.
Department of Orthodontics and Craniofacial Biology

Website address:
www.orthodontics.nl

11 Review team members and their organisational affiliations
Give the title, first name and last name of all members of the team working directly on the review. Give the organisational affiliations of each member of the review team.

Title	First name	Last name	Affiliation
Ms	Mette	Kuijpers	Department of Orthodontics and Craniofacial Biology, Radboud University Nijmegen Medical Centre
Ms	yu-Ting	Chiu	
Dr	Piotr	Fudalej	Department of Orthodontics and Craniofacial Biology, Radboud University Nijmegen Medical Centreen

12 Funding sources/sponsors
Give details of the individuals, organizations, groups or other legal entities who take responsibility for initiating, managing, sponsoring and/or financing the review. Any unique identification numbers assigned to the review by the individuals or bodies listed should be included.
no external funding

13 Conflicts of interest
List any conditions that could lead to actual or perceived undue influence on judgements concerning the main topic investigated in the review.
Are there any actual or potential conflicts of interest?
None known

14 Collaborators
Give the name, affiliation and role of any individuals or organisations who are working on the review but who are not listed as review team members.

Title	First name	Last name	Organisation details

Review methods

15 Review question(s)
State the question(s) to be addressed / review objectives. Please complete a separate box for each question.
To identify 3D imaging methods for quantitative assessment of soft tissue and skeletal morphology in patients with cleft lip and palate.

16 Searches
Give details of the sources to be searched, and any restrictions (e.g. language or publication period). The full search strategy is not required, but may be supplied as a link or attachment.
To identify publications, a literature search up to December 2011 was performed in PubMed (1948-2011), EMBASE (1980-2011), Scopus (2004-2011), Web of Science (1945-2011) and Cochrane Library. The list of terms was developed and databases were selected with the help of a senior librarian who specialized in health sciences. The terms used in the search strategy were: 1-Concerning cleft lip and palate: cleft lip, cleft palate, CLP, UCLP, BCLP 2-Three dimensional: Imaging Three-Dimensional, 3D, three dimensional, Image, images, imaging, 3D image, 3D images, 3D imaging, 3-CT: Tomography, X-Ray Computed, Computed Tomographic, CT, volumetric CT, computed tomography, computer assisted tomography 4-CBCT: Cone Beam Computed Tomography, CBCT, Spiral Cone Beam Computed Tomography 5- Photos: Photogrammetry, stereophotogrammetry 6-MRI: Magnetic Resonance Imaging, Magnetic Resonance Image, Magnetic Resonance Images, MRI 7-4D: 4D, 4-dimensional, Four Dimensional Computed Tomography 8-Ultrasound: ultrasonography, echography

17 URL to search strategy

If you have one, give the link to your search strategy here. Alternatively you can e-mail this to PROSPERO and we will store and link to it.

18 Condition or domain being studied

Give a short description of the disease, condition or healthcare domain being studied. This could include health and wellbeing outcomes.

For evaluating results of cleft lip and palate treatment, record taking is proposed at certain ages. However, both Eurocleft and Americleft cannot give strict guidelines. Final decisions for treatment protocols and timing of records taking still depend on each cleft palate team. With latest 3D imaging technology introduced, it is expected that a majority of teams is using these newer techniques to assess their treatment results.

19 Participants/population

Give summary criteria for the participants or populations being studied by the review. The preferred format includes details of both inclusion and exclusion criteria.

Patients with non-syndromic cleft lip and palate over 5 years of age.

20 Intervention(s), exposure(s)

Give full and clear descriptions of the nature of the interventions or the exposures to be reviewed

3D imaging methods.

21 Comparator(s)/control

Where relevant, give details of the alternatives against which the main subject/topic of the review will be compared (e.g. another intervention or a non-exposed control group).

Not applicable.

22 Types of study to be included initially

Give details of the study designs to be included in the review. If there are no restrictions on the types of study design eligible for inclusion, this should be stated.

Primary publications using 3D imaging techniques with quantitative assessing results of facial soft tissue or skeletal morphology in patients with cleft lip/palate

23 Context

Give summary details of the setting and other relevant characteristics which help define the inclusion or exclusion criteria.

24 Primary outcome(s)

Give the most important outcomes.

Reliability of the quantitative results from different 3D imaging methods

Give information on timing and effect measures, as appropriate.

Not applicable.

25 Secondary outcomes

List any additional outcomes that will be addressed. If there are no secondary outcomes enter None.

none

Give information on timing and effect measures, as appropriate.

Not applicable.

26 Data extraction, (selection and coding)

Give the procedure for selecting studies for the review and extracting data, including the number of researchers involved and how discrepancies will be resolved. List the data to be extracted.

27 Risk of bias (quality) assessment

State whether and how risk of bias will be assessed, how the quality of individual studies will be assessed, and whether and how this will influence the planned synthesis.

The included studies are evaluated according to the quality assessment instrument (QAI) used by Gordon et al. (2009) I. Study design (7 ?) A. Objective—objective clearly formulated (?) B. Sample size—considered adequate (?) C. Sample size—estimated before collection of data (?) D. Selection criteria—clearly described (?) E. Baseline characteristics—similar baseline characteristics (?) F. Timing—prospective (?) G. Randomization—stated (?) II. Study

measurements (3 ?) H. Measurement method—appropriate to the objective (?) I. Blind measurement—blinding (?) J. Reliability—adequate level of agreement (?) III. Statistical analysis (5 ?) K. Dropouts—dropouts included in data analysis (?) L. Statistical analysis—appropriate for data (?) M. Confounders—confounders included in analysis (?) N. Statistical significance level—P value stated (?) O. Confidence intervals provided (?) Maximum number of ?s = 15 A checkmark is scored when a criterion is fulfilled. Depending on the study design a quality assessment is performed on a maximum of 15 criteria. Study quality is expressed as the percentage of criteria fulfilled in relation to the total number of applicable criteria. The score per study is calculated as a percentage by dividing the number of checkmarks by the number of applicable criteria and multiplying by 100. In cases where criteria are not applicable to the study design, the scoring is marked as a dot and the overall percentage score is based on the valid criteria for that study design.

28 Strategy for data synthesis

Give the planned general approach to be used, for example whether the data to be used will be aggregate or at the level of individual participants, and whether a quantitative or narrative (descriptive) synthesis is planned. Where appropriate a brief outline of analytic approach should be given.

Data will be analyzed separately for each 3D imaging tool, study details will be pooled into tables and a descriptive summary will be listed. This will also assist in understanding how the 3D imaging tool is used nowadays in quantitative assessment of soft tissue and skeletal morphology in patients with cleft lip and palate.

29 Analysis of subgroups or subsets

Give any planned exploration of subgroups or subsets within the review. ‘None planned’ is a valid response if no subgroup analyses are planned.

none

Review general information

30 Type of review

Select the type of review from the drop down list.

Diagnostic

31 Language

Select the language(s) in which the review is being written and will be made available, from the drop down list. Use the control key to select more than one language.

English

Will a summary/abstract be made available in English?

Yes

32 Country

Select the country in which the review is being carried out from the drop down list. For multi-national collaborations select all the countries involved. Use the control key to select more than one country.

Netherlands

33 Other registration details

List places where the systematic review title or protocol is registered (such as with he Campbell Collaboration, or The Joanna Briggs Institute). The name of the organisation and any unique identification number assigned to the review by that organization should be included.

34 Reference and/or URL for published protocol

Give the citation for the published protocol, if there is one.

Give the link to the published protocol, if there is one. This may be to an external site or to a protocol deposited with CRD in pdf format.

35 Dissemination plans

Give brief details of plans for communicating essential messages from the review to the appropriate audiences.

Do you intend to publish the review on completion?

Yes

36 Keywords

Give words or phrases that best describe the review. (One word per box, create a new box for each term)

37 Details of any existing review of the same topic by the same authors

Give details of earlier versions of the systematic review if an update of an existing review is being registered, including full bibliographic reference if possible.

38 Current review status

Review status should be updated when the review is completed and when it is published.

Ongoing

39 Any additional information

Provide any further information the review team consider relevant to the registration of the review.

40 Details of final report/publication(s)

This field should be left empty until details of the completed review are available.
Give the full citation for the final report or publication of the systematic review.
Give the URL where available.
